# Accumulation of Kaempferitrin and Expression of Phenyl-Propanoid Biosynthetic Genes in Kenaf (*Hibiscus cannabinus*)

**DOI:** 10.3390/molecules191016987

**Published:** 2014-10-23

**Authors:** Shicheng Zhao, Xiaohua Li, Dong Ha Cho, Mariadhas Valan Arasu, Naif Abdullah Al-Dhabi, Sang Un Park

**Affiliations:** 1 Department of Crop Science, Chungnam National University, 99 Daehak-Ro, Yuseong-Gu, Daejeon 305-764, Korea; E-Mails: zhaoshicheng@msn.com (S.Z.); lixiaohua2007@hotmail.com (X.L.); 2 College of Biomedical Science, Kangwon National University, Chuncheon 200-701, Korea; E-Mail: chodh@kangwon.ac.kr; 3 Department of Botany and Microbiology, Addiriyah Chair for Environmental Studies, College of Science, King Saud University, P.O. Box 2455, Riyadh 11451, Saudi Arabia; E-Mail: mvalanarasu@gmail.com; 4 Visiting Professor Program, King Saud University, P.O. Box 2455, Riyadh 11451, Saudi Arabia

**Keywords:** flavone synthase, kaempferitrin, kenaf, phenylpropanoid biosynthesis

## Abstract

Kenaf (*Hibiscus cannabinus*) is cultivated worldwide for its fiber; however, the medicinal properties of this plant are currently attracting increasing attention. In this study, we investigated the expression levels of genes involved in the biosynthesis of kaempferitrin, a compound with many biological functions, in different kenaf organs. We found that phenylalanine ammonia lyase (*HcPAL*) was more highly expressed in stems than in other organs. Expression levels of cinnamate 4-hydroxylase (*HcC4H*) and 4-coumarate-CoA ligase (*Hc4CL*) were highest in mature leaves, followed by stems and young leaves, and lowest in roots and mature flowers. The expression of chalcone synthase (*HcCHS*), chalcone isomerase (*HcCHI*), and flavone 3-hydroxylase (*HcF3H*) was highest in young flowers, whereas that of flavone synthase (*HcFLS*) was highest in leaves. An analysis of kaempferitrin accumulation in the different organs of kenaf revealed that the accumulation of this compound was considerably higher (>10-fold) in leaves than in other organs. On the basis of a comparison of kaempferitrin contents with the expression levels of different genes in different organs, we speculate that *HcFLS* plays an important regulatory role in the kaempferitrin biosynthetic pathway in kenaf.

## 1. Introduction

**Scheme 1:**
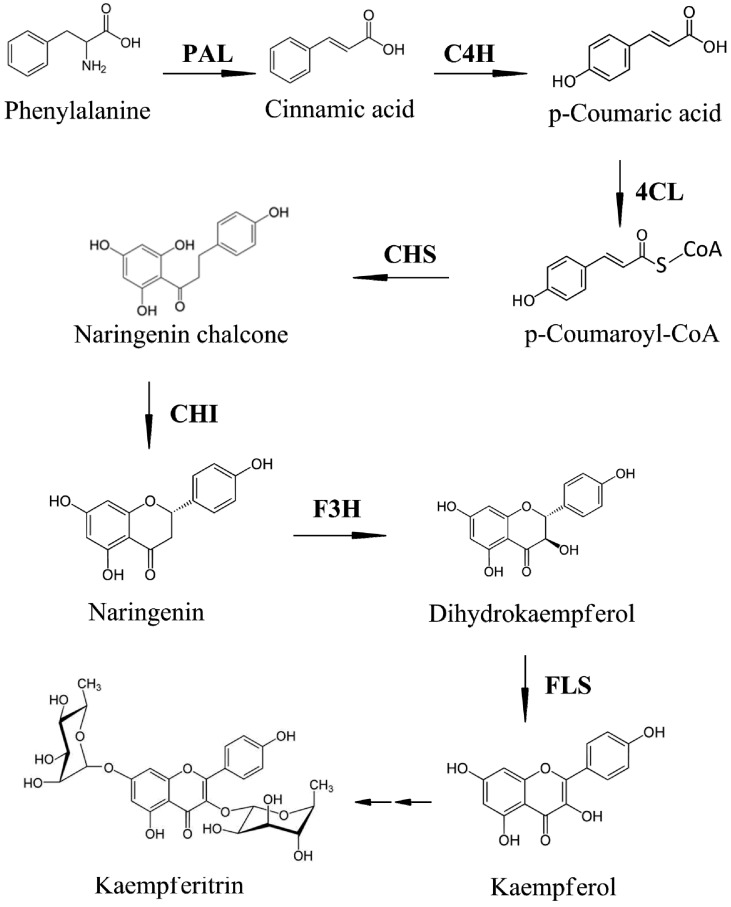
Schematic of kaempferitrin biosynthesis in *Hibiscus cannabinus*.

PAL, phenylalanine ammonia lyase; C4H, cinnamic acid 4-hydroxylase; 4CL, 4-coumarate-CoA ligase; CHS, chalcone synthase; CHI, chalcone isomerase; F3H, flavanone-3-hydroxylase; FLS, flavonol synthase.

Kaempferol, a flavonoid that has been isolated from many plants [1,2,3,4], has many important health-promoting effects, such as antioxidant, antimicrobial, and anti-inflammatory effects, and is known to decrease the risk of cancers, including bladder, colon, and squamous cell cancers [5,6,7,8,9]. Flavonoid glycosides are known to be more abundant than their flavonoid monomers in plants and exhibit a broad range of biological activities. One such flavonoid glycoside, kaempferitrin (kaempferol 3,7-dirhamnoside), has been shown to have a significant hypoglycemic effect and antioxidant properties in humans and is one of the effect factors of auxin transport in plants [10,11]. Biosynthesis of kaempferitrin is one of the sub-pathways of phenylpropanoid biosynthesis. Phenylpropanoids are a diverse group of compounds involved in plant defense, structural support, and survival [12]. Phenylpropanoid biosynthesis starts with the formation of the aromatic amino acid phenylalanine, which is in turn converted into cinnamic acid by phenylalanine ammonia lyase (PAL), the first enzyme in the phenylpropanoid pathway. Cinnamate 4-hydroxylase (C4H) subsequently catalyzes trans-cinnamic acid hydroxylate into *p*-coumaric acid, which is then catalyzed to *p*-coumaroyl-CoA by 4-coumarate-CoA ligase (4CL). *p*-coumaroyl-CoA is the precursor for many phenylpropanoid products, such as lignins and flavonoids. Subsequently, chalcone synthase (CHS) catalyzes the production of naringenin chalcone, the precursor for all flavonoids, and in the next step, chalcone isomerase (CHI) converts naringenin chalcone to naringenin. Thereafter, naringenin is converted to dihydrokaempferol by flavone 3-hydroxylase (F3H), and flavone synthase (FLS) catalyzes the conversion of dihydrokaempferol into kaempferol. In the final step, kaempferitrin is synthesized via two biosynthetic intermediates [13] (Scheme 1).

Kenaf (*Hibiscus cannabinus*), which is a member of the Malvaceae family, is a fast-growing annual herbaceous dicotyledenous plant that is widely distributed from temperate to tropical areas [14]. On account of its stem fibers, the plant has been used for thousands of years in the manufacture of flax. Nowadays, the uses of kenaf have diversified into many areas, such as the pulp and paper industry, soil remediation, biofuel production, and seed oil utilization [15,16,17,18]. During the last decades, some studies have indicated that kenaf exhibits a vast array of important medicinal properties, including anticancer, antioxidant, and hepatoprotective activities [19,20,21]. Most of the medicinal benefits attributed to kenaf are due to the presence of phenylpropanoid compounds in this plant. The genes that encode phenylpropanoid biosynthetic enzymes have been extensively characterized in maize, petunia, and *Arabidopsis* [22,23,24]. Furthermore, in kenaf, the genes that encode phenylpropanoid biosynthetic enzymes such as *HcPAL*, *Hc4CL*, *HcC4H*, and *HcCHS* have been isolated in previous studies [25,26,27].

However, there is no information available on gene expression in the phenylpropanoid biosynthetic pathway in kenaf. Additionally, although some studies have investigated the contents of a few phenylpropanoids in the leaves or seeds of kenaf [20,28], studies on the phenolic contents of different organs in kenaf are generally very limited. Thus, in this study, we aimed to investigate the expression of phenylpropanoid biosynthetic genes and kaempferol and kaempferitrin accumulation in different organs (roots, stems, young and mature leaves, and young and mature flowers) of kenaf. The findings of this study will contribute to the theoretical basis for further studies on the genetic engineering and medicinal value of kenaf.

## 2. Results and Discussion

### 2.1. Expression of Phenylpropanoid Biosynthetic Genes in Kenaf

**Figure 1:**
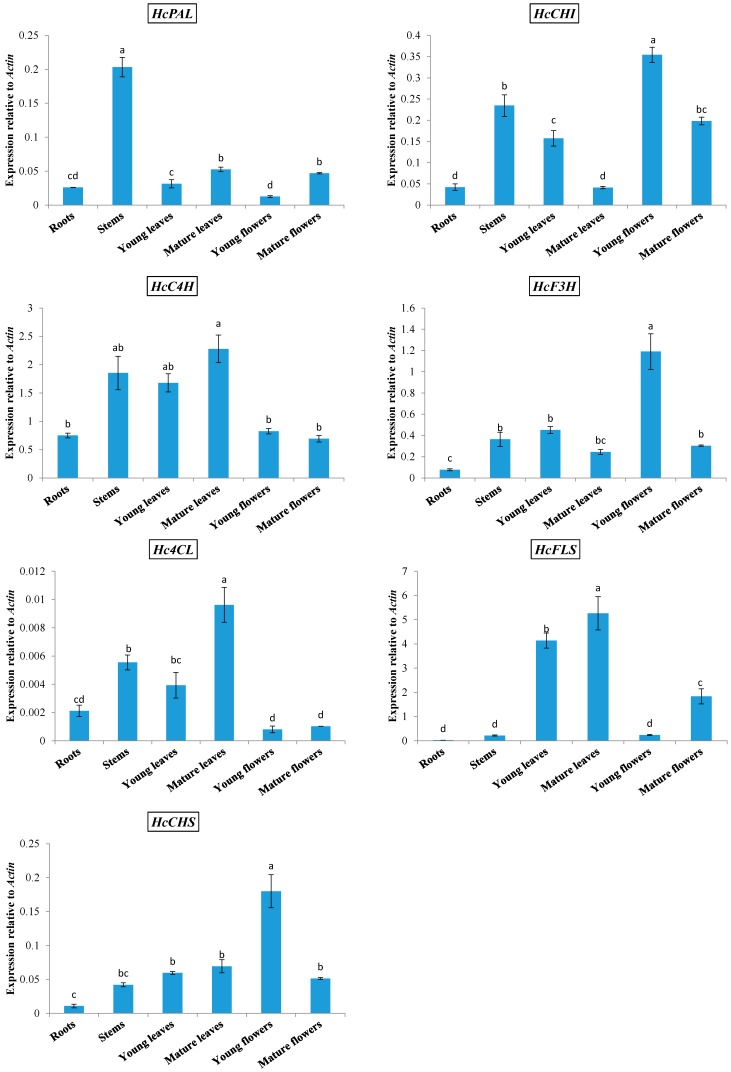
Expression of phenylpropanoid biosynthetic genes in different organs of *Hibiscus cannabinus*.

The expression of phenylpropanoid biosynthetic genes was analyzed in the roots, stems, young and mature leaves, and young and mature flowers of kenaf by qRT-PCR (Figure 1). Three phenylpropanoid biosynthetic genes (*HcPAL*, *HcC4H,* and *Hc4CL*) were expressed at relatively high levels in stems. The main product of kenaf stems is fiber, which is composed of cellulose and lignin, the latter of which is a phenylpropanoid compound. The third metabolite in the phenylpropanoid biosynthetic pathway is *p*-coumaroyl-CoA, which is the precursor for many phenylpropanoid products, and we speculate that phenylpropanoid biosynthesis in stems is channeled mainly into the lignin branch. The expression levels of *HcC4H* and *Hc4CL* were highest in mature leaves, followed by stems and young leaves, but were low in roots, young flowers, and mature flowers. The expression of *HcCHS*, *HcCHI,* and *HcF3H* was higher in young flowers compared with that in other organs. *HcFLS* was expressed at the highest levels in young and mature leaves, was moderately expressed in mature flowers, and expressed at very low levels in roots, stems, and young flowers.

On the basis of previous studies by our group, we know that the expression of different phenylpropanoid biosynthetic genes in different organs is species-specific [1,2,4,29,30]. For example, in *Prunella vulgaris* [29] and *Lycium chinense* [4], the highest expression of the first enzyme-coding gene in the phenylpropanoid pathway *PAL* was observed in flowers. However, in *Fagopyrum esculentum* [1] and *Allium sativum* [2], the expression of *PAL* was highest in roots, and was also highly expressed in the stems of *F. esculentum*. Similarly, in *Scutellaria baicalensis,* the highest and lowest expressions of FLS were observed in the roots and leaves, respectively [30]. In direct contrast, in *L. chinense*, the expression of *FLS* was highest in leaves and lowest in roots.

Each value is the mean of three replicates, and error bars indicate standard deviations. The different letter in same line means significant difference (*p* < 0.05).

### 2.2. Kaempferitrin Content in Different Organs of Kenaf

**Figure 2:**
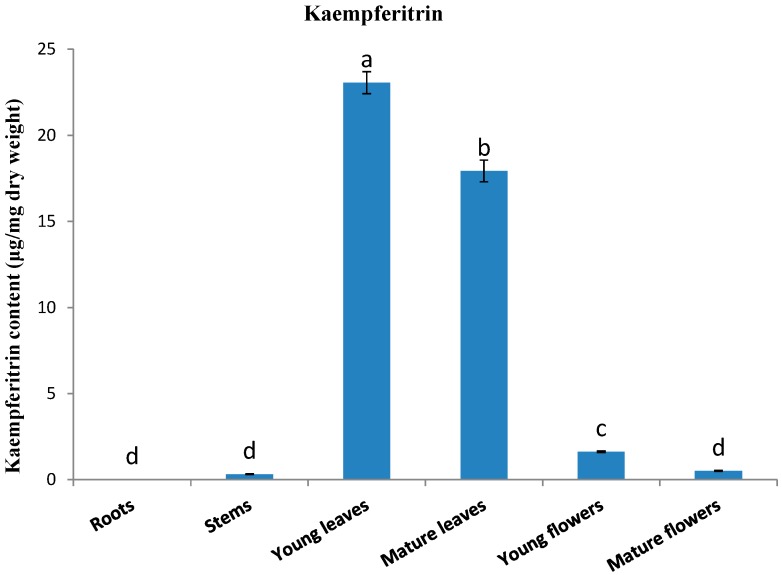
Accumulation of kaempferitrin in different organs of *Hibiscus cannabinus*.

Figure 2 shows the kaempferitrin contents in the different organs of kenaf. Large amounts of kaempferitrin were detected in young leaves [23.05 μg/mg dry weight (DW)] and mature leaves (17.93 μg/mg DW). In contrast, relatively small amounts were detected in young flowers (1.62 μg/mg DW), mature flowers (0.51 μg/mg DW), and stems (0.32 μg/mg DW), and no kaempferitrin was detected in roots. Similar results were obtained for *Siraitia grosvenorii* [31], with the highest content being detected in the leaves (17.62 μg/mg DW) and lowest content in the stems (1.67 μg/mg DW). Rho *et al.* [28] recorded a kaempferitrin concentration of 29.3 μg/mg DW in leaf extracts of kenaf. However, after treating the kenaf leaf extracts with far-infrared (FIR) irradiation for 1 h, certain deglycosylation products (kaempferol, 15.2 μg/mg DW; afzelin, 2.4 μg/mg DW; and α-rhamnoisorobin, 5.7 μg/mg DW) were detected, and the content of kaempferitrin was decreased (3.1 μg/mg DW). These authors also found that the deglycosylation products can enhance protection against UV irradiation by inhibiting the activity of tyrosinase. Pinheiro *et al.* [32] examined the amounts of kaempferitrin in the leaves of *Bauhinia forficate* plants growing in two regions of Brazil (Telemaco Borba and Itajai). They found that whereas leaf extracts from plants growing in Itajai had a kaempferitrin content of 1952.59 μg/mg, those from plants growing in Telemaco Borba contained just 211.61 μg/mg. This finding emphasizes that the production of *O*-glycosylflavonoid derivatives can show wide variability, depending on environmental conditions.

Each value is the mean of three replicates, and error bars indicate standard deviations. The different letter in same line means significant difference (*p* < 0.05).

With regard to the expression of phenylpropanoid biosynthetic genes in different organs, that of *HcFLS* was highly consistent with the content of kaempferitrin, being highly expressed in young and mature leaves, and poorly expressed in stems, roots, and young and mature flowers. On the basis of the highly consistent *HcFLS* expression and kaempferitrin content in different organs, we suggest that kaempferitrin biosynthesis is mainly controlled by the gene *HcFLS*, in the kaempferitrin biosynthetic pathway.

## 3. Experimental Section

### 3.1. Plant Materials

Seeds of *Hibiscus cannabinus* were germinated in a growth chamber, after which the seedlings were transferred to the experimental farm at Kangwon National University, Chunchon, Korea. The flowers, leaves, stems and roots were excised from mature plants. The samples were immediately frozen in liquid nitrogen and then stored at −80 °C and/or freeze-dried for RNA isolation and/or HPLC analysis.

### 3.2. Chemicals and Solvents

For the chemical analysis of kaempferitrin, the external standard kaempferol 3,7-dirhamnoside was purchased from Extrasynthese (Genay, France). HPLC-grade methanol (CH_3_OH) was purchased from J. T. Baker Chemical Co. (Phillipsburg, NJ, USA), and acetic acid was provided by Kanto Chemical Co., Inc. (Tokyo, Japan).

### 3.3. RNA Extraction and cDNA Synthesis

Total RNA was isolated from the different organs of *H. cannabinus* following the method described by Ghosh *et al.* [33]. Briefly, 1 g of organ tissue was ground in liquid nitrogen using a mortar and pestle. The resulting powder was transferred to a pre-cooled 15-mL tube, to which 5 mL of pre-warmed CTAB extraction buffer containing 2% (w/v) 2-mercaptoethanol were immediately added. The tube contents were mixed vigorously, and incubated at 65 °C for 20 min. After cooling, the 5 mL of chloroform was added, mixed well, and centrifuged at 8000× *g* for 30 min. The supernatant was transferred to a new tube and the chloroform extraction step was repeated. A one-third volume of DEPC-treated 8 M LiCl was added, followed by incubation overnight at 4 °C and centrifugation at 8000× *g* and 4 °C for 1 h. The RNA pellet was dissolved in 500 μL Qiagen buffer RLT (RNeasy Mini kit, Qiagen, Valencia, CA, USA), washed, and eluted two times with 50 μL DEPC water following the manufacturer’s protocol. The RNA integrity was checked by running on an agarose gel. Having extracted the total RNAs of different organs, the concentrations were determined by spectrophotometric analysis (NanoVue Plus; GE Healthcare, Buckinghamshire, UK). For first-strand cDNA synthesis, 1 μg of high-quality total RNA was used for reverse transcription (RT) using a ReverTra Ace-kit (Toyobo Co. Ltd., Osaka, Japan). A 20-fold dilution of 20 μL of the resulting cDNA was used as a template for quantitative real-time PCR (qRT-PCR).

### 3.4. QRT-PCR Analysis

On the basis of the published gene sequences of *HcPAL*, *HcC4H*, *Hc4CL*, *HcCHS*, *HcCHI*, *HcF3H*, and *HcFLS* (GenBank accession numbers JQ779022, JX524279, HM151379, KJ523130, KJ523131, KJ523132 and KJ523133, respectively), qRT-PCR primers were designed using the Primer 3 website [34,35] (Table 1). The expression of these genes was determined using the relative quantification method, with the *Actin* housekeeping gene (accession number EU048368) of *H. cannabinus* used as a reference. For quantification of the standard, PCR products amplified from cDNA were purified, and the concentration of the products was measured in order to calculate the number of cDNA copies. QRT-PCR reactions were performed in a 20-μL reaction mixture, containing 5 μL of template cDNA, 10 μL of 1 × SYBR Green Real-time PCR Master Mix (Toyobo Co. Ltd.), 0.5 μL of each primer, and DEPC-treated water. Thermal cycling conditions were as follows: 95 °C for 15 min, followed by 40 cycles of 95 °C for 20 s, 53 °C for 40 s, and 72 °C for 30 s. The PCR reactions were performed in a CFX96 Real-Time system (Bio-Rad, Hercules, CA, USA), and the PCR products were analyzed using Bio-Rad CFX Manager 2.0 software. Three replicates of each sample were used for the qRT-PCR analysis, and the values are expressed as means ± SDs.

**Table 1:** Primers used for real-time PCR.

Primer	Sequence (5' to 3')	Amplicon (Base Pairs)
HcPAL F	ACAGGAAGCCTTTCGTCTAGC	218
HcPAL R	TATGTGTCAAGTGGTCGGTGA	
HcC4H F	GTAGATTGGCACAGAGCTTCG	198
HcC4H R	CGATGGCACATTTAAGAGCAT	
Hc4CL F	TGAATTCATCTTTCGGTCCAG	221
Hc4CL R	GCATGATGACATCTCCCTGTT	
HcCHS F	GGGCTTACATTTCACCTCCTC	225
HcCHS R	GTTCCCGTATTCCGAAAGAAC	
HcCHI F	GTAAGAAAGCCGAGGAGTTGG	190
HcCHI R	GCCTTTTCTTCTGCATCAGTG	
HcF3H F	TGTGTGGACATGGATCAGAAA	214
HcF3H R	GATCTCCAAGGTTGACCACAA	
HcFLS F	AACCAGATGTGCTGAAACAGG	239
HcFLS R	CTGGTCGCCAATGTGAATAAT	
HcActin F	GTCTAGACCTTGCTGGTCGTG	210
HcActin R	CTTGTCCATCAGGCAATTCAT	

### 3.5. Extraction and Quantification of Kaempferitrin

Different organs of *H. cannabinus* were freeze-dried at −80 °C for 48 h, and then ground to a fine powder using a mortar and pestle. Kaempferitrin was extracted from the *H. cannabinus* samples (0.02 g) by adding methanol (3 mL) of containing 0.1% ascorbic acid (w/v) at 60 °C for 1 h. After centrifuging the extract (3000 rpm), the supernatant was filtered using a 0.22-μm Acrodisc syringe filter (Pall Corp., Port Washington, NY, USA), and analyzed by HPLC. Kaempferitrin were separated on a C_18_ column (250 × 4.6 mm, 5 μm; RStech, Daejeon, Korea) using an Agilent 1100 HPLC system (Agilent Technologies France, Massy, France) equipped with a photodiode array detector (Supplementary Figure S1). The mobile phase consisted of a mixture of (A) MeOH/water/acetic acid (5:92.5:2.5, v/v/v) and (B) MeOH/water/acetic acid (95:2.5:2.5, v/v/v), using a previously described procedure [36]. The initial mobile phase composition was 0% solvent B, followed by a linear gradient from 0% to 80% of solvent B in 48 min, then holding at 0% solvent B for an additional 10 min, and the column was maintained at 30 °C. The flow rate was maintained at 1.0 mL/min, the injection volume was 20 μL, and the detection wavelength was 280 nm. The concentrations of compounds were determined using a standard curve. All samples were analyzed in triplicate, and the values are expressed as means ± SDs.

## 4. Conclusions

In this study, we investigated the accumulation of kaempferitrin and expression of phenylpropanoid biosynthetic pathway-related genes in different kenaf organs. All genes investigated were expressed at very low levels in roots, whereas each gene showed different expression patterns in different organs. From an analysis of compounds, we observed that leaves contain large amounts of kaempferitrin. On the basis of a comparison of kaempferitrin content with different gene expression patterns in different organs, we speculate that kaempferitrin biosynthesis is mainly controlled by the *FLS* gene. Our results may help us to understand the molecular mechanisms underlying the biosynthesis of kaempferitrin in *H. cannabinus*. Furthermore, our study indicates that people should use the leaves of *H. cannabinus* to maximize the benefits of kaempferitrin.

## Supplementary Materials

Supplementary materials can be accessed at: http://www.mdpi.com/1420-3049/19/10/16987/s1.

## Supplementary Files

Supplementary File 1
