# Supplementary material for: Accumulation of Kaempferitrin and Expression of Phenyl-Propanoid Biosynthetic Genes in Kenaf (Hibiscus cannabinus)
Source: Molecules. 2014 Oct 23;19(10):16987–97. doi: 10.3390/molecules191016987 (PMC6270828; doi:10.3390/molecules191016987)

Supplementary Material

Figure S1. Comparative HPLC chromatograms of *Hibiscus cannabinus*. Chromatograms showing reference standard (**A**) and methanol extract (**B**) of *H. cannabinus* young leaves.

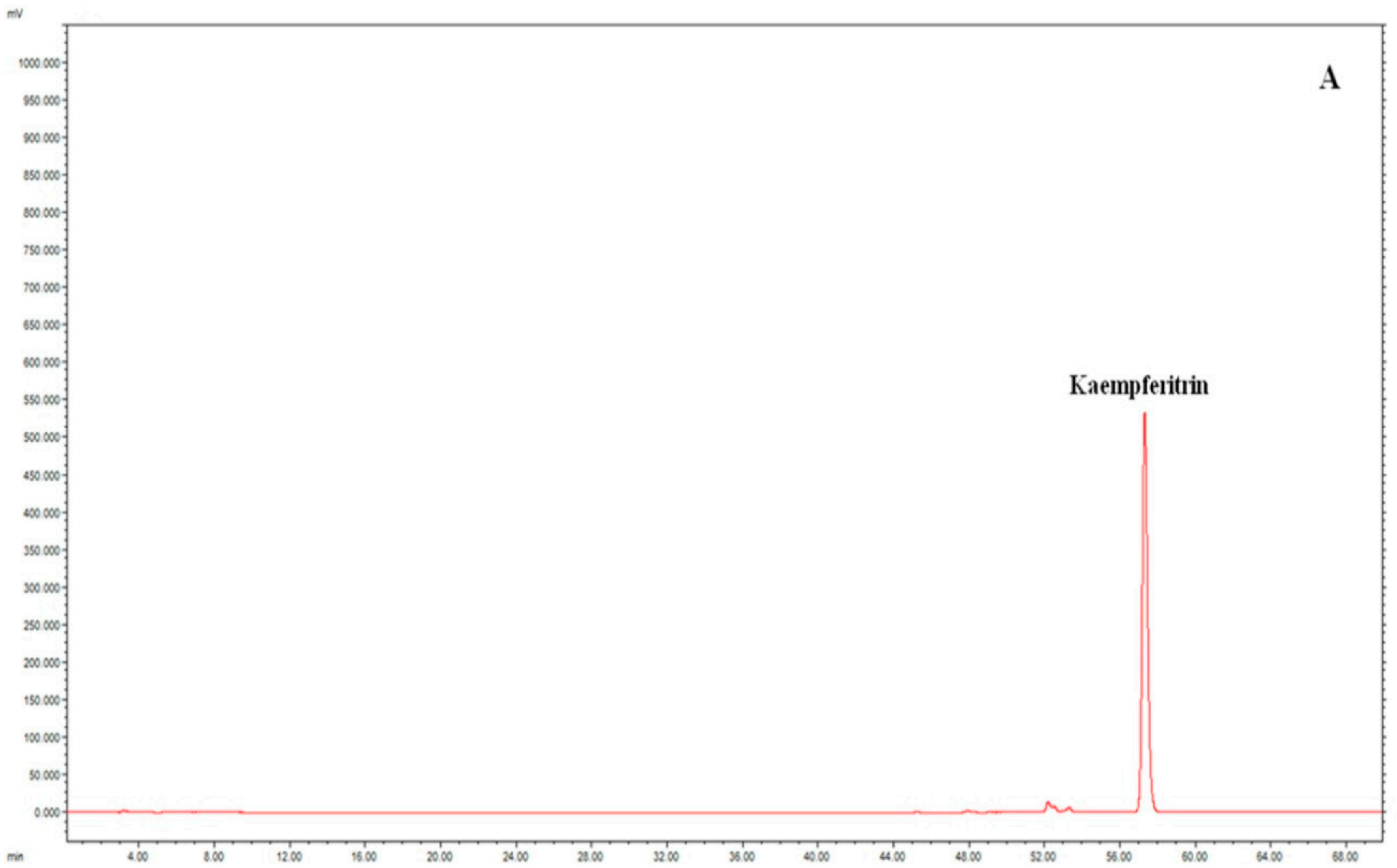

Figure S1. *Cont.*

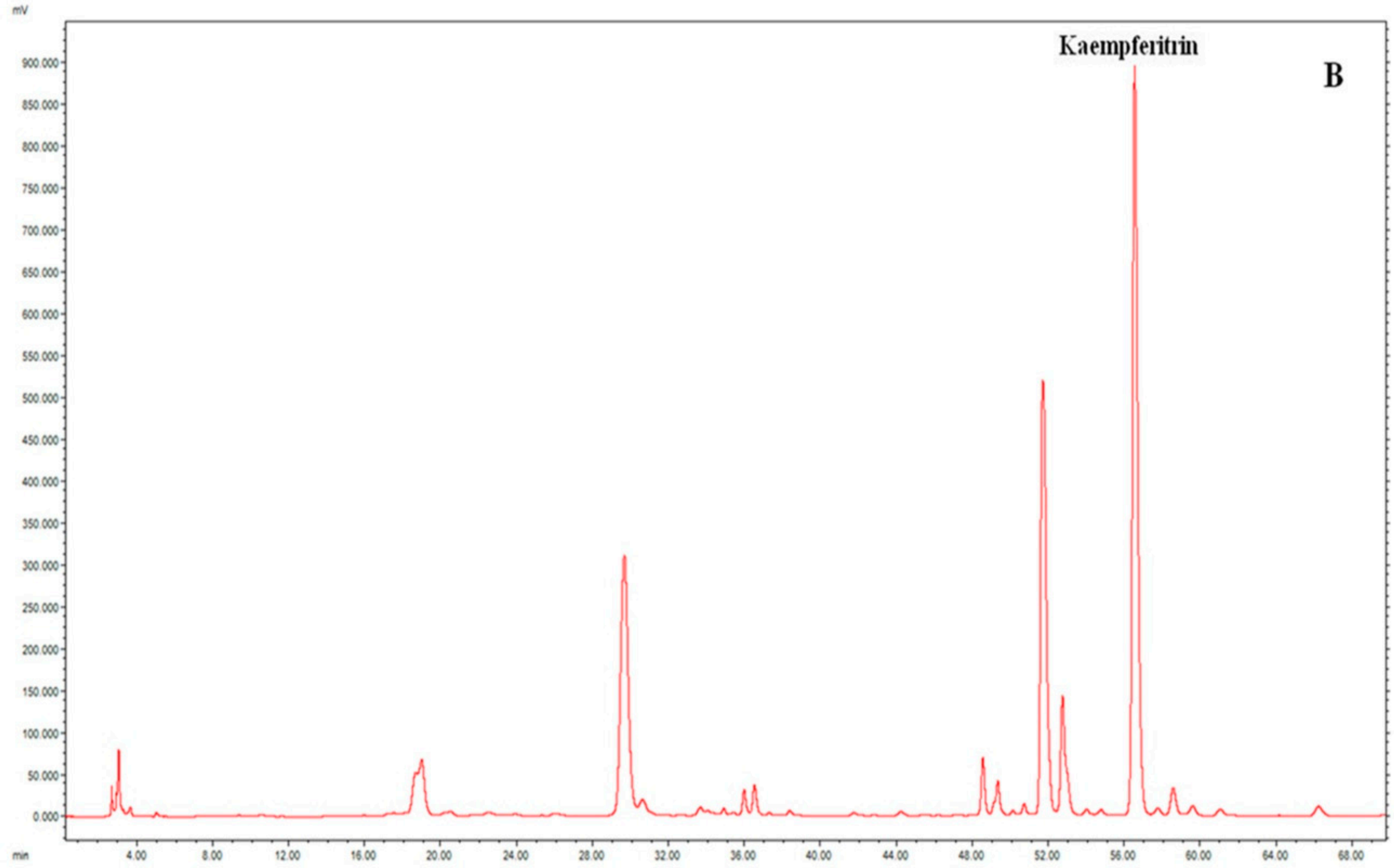

Supplement: Supplementary File 1 [file molecules-19-16987-s001.pdf]
